# Effect of Glucose Improvement on Nocturnal Sleep Breathing Parameters in Patients with Type 2 Diabetes: The Candy Dreams Study

**DOI:** 10.3390/jcm9041022

**Published:** 2020-04-04

**Authors:** Liliana Gutiérrez-Carrasquilla, Carolina López-Cano, Enric Sánchez, Ferran Barbé, Mireia Dalmases, Marta Hernández, Angela Campos, Anna Michaela Gaeta, Paola Carmona, Cristina Hernández, Rafael Simó, Albert Lecube

**Affiliations:** 1 Endocrinology and Nutrition Department, University Hospital Arnau de Vilanova, Obesity, Diabetes and Metabolism (ODIM) research group, IRBLleida, University of Lleida, 25198 Lleida, Spain; liligutierrezc@gmail.com (L.G.-C.); karolopezc@gmail.com (C.L.-C.); esanchez@irblleida.cat (E.S.); martahernandezg@gmail.com (M.H.); angelacamposjimenez@gmail.com (A.C.); 2 Respiratory Department, University Hospital Arnau de Vilanova-Santa María, Translational Research in Respiratory Medicine, IRBLleida, University of Lleida, 25198 Lleida, Spain; febarbe.lleida.ics@gencat.cat (F.B.); mdalmases.lleida.ics@gencat.cat (M.D.); annamichelagaeta@hotmail.it (A.M.G.); pcarmona@irblleida.cat (P.C.); 3 Centro de Investigación Biomédica en Red de Enfermedades Respiratorias (CIBERES), Instituto de Salud Carlos III (ISCIII), 28029 Madrid, Spain; 4 Endocrinology and Nutrition Department, University Hospital Vall d’Hebron, Diabetes and Metabolism Research Unit, Vall d’Hebron Institut de Recerca (VHIR), Autonomous University of Barcelona, 08035 Barcelona, Spain; cristina.hernandez@vhir.org; 5 Centro de Investigación Biomédica en Red de Diabetes y Enfermedades Metabólicas Asociadas (CIBERDEM), Instituto de Salud Carlos III (ISCIII), 28029 Madrid, Spain

**Keywords:** diabetes, apnea, hypoxia, glycated hemoglobin

## Abstract

Type 2 diabetes exerts a negative impact on sleep breathing. It is unknown whether a long-term improvement in glycemic control ameliorates this effect. We conducted an interventional study with 35 patients with type 2 diabetes and obstructive sleep apnea (OSA) to explore this. At home, sleep breathing parameters were assessed at baseline and after a 4-month period in which antidiabetic therapy was intensified. Patients who decreased their body mass index ≥2kg/m^2^ were excluded. Those with an HbA1c reduction ≥0.5% were considered good responders (*n* = 24). After the follow-up, good responders exhibited an improvement in the apnea–hypopnea index (AHI: 26-1 (95% IC: 8.6–95.0) vs. 20.0 (4.0–62.4) events/hour, *p* = 0.002) and in time with oxygen saturation below 90% (CT90: 13.3 (0.4–69.0) vs. 8.1 (0.4–71.2) %, *p* = 0.002). No changes were observed in the group of non–responders (*p* = 0.722 and *p* = 0.138, respectively). The percentage of moderate and severe OSA decreased among good responders (*p* = 0.040). In the wider population, the change in HbA1c correlated positively to decreases in AHI (r = 0.358, *p* = 0.035) and negatively to increases in the minimum arterial oxygen saturation (r = −0.386, *p* = 0.039). Stepwise multivariate regression analysis showed that baseline AHI and the absolute change in HbA1c independently predicted decreased AHI (R^2^ = 0.496). The improvement of glycemic control exerts beneficial effects on sleep breathing parameters in type 2 diabetes, which cannot be attributed merely to weight loss.

## 1. Introduction

In recent years, increasing evidence has appeared to support the idea that type 2 diabetes might contribute to the development of sleep breathing disorders characterized by sleep fragmentation and intermittent hypoxia [1]. In fact, patients with type 2 diabetes exhibit atypical and reduced ventilatory responses to isocapnic hypoxia, being powerless to respond in a homeostatic manner to a hypoxic challenge [2]. Thus, the Sweet Sleep Study showed how the same apnea–hypopnea index (AHI) in patients with type 2 diabetes was characterized by reduced hypopnea events and increased apnea episodes in comparison to subjects without diabetes, resulting in a more severe sleep breathing pattern [3]. Consequently, patients with type 2 diabetes spend three to four times more of their sleeping time with oxygen saturation below 90% (CT90) of the population without diabetes [4]. Moreover, type 2 diabetes is an independent risk factor for excessive daytime sleepiness and almost two out of every three patients are categorized as “poor sleepers” [5]. This is a crucial finding because sleep-related hypoxemia and sleep fragmentation lead to sympathetic overactivity, endothelial dysfunction, an atherogenic lipid profile and increased blood pressure, factors that may contribute to the enhanced cardiovascular risk detected in type 2 diabetes [6,7]. Insulin resistance, chronic hyperglycemia, low-grade inflammation and leptin resistance in central respiratory control might act as mediators for the development of sleep breathing disorders in type 2 diabetes [1]. In addition, the presence of some degree of diabetic autonomic neuropathy, with a powerlessness to respond appropriately to nocturnal airflow reductions, has been also implicated in this relationship [8]. 

The amelioration of sleep breathing disorders in patients with type 2 diabetes through better metabolic control has been only partially examined. In a case-control study involving 30 patients with type 2 diabetes and 10 control subjects, a 5-day hospital admission period of glucose optimization achieved a significant reduction in nocturnal oxygen desaturation events [9]. However, it is unknown whether a long-term improvement in glycemic control with type 2 diabetes can also ameliorate their sleep breathing parameters. In this setting, changes in body weight appear as a major confounding factor. Abdominal obesity is the main risk factor for obstructive sleep apnea and yet moderate reductions in body weight are associated with marked reductions in the apnea–hypopnea index [10].

In order to shed light on this issue, we present an interventional study aimed at examining whether the improvement of metabolic control during a three-month period in patients with type 2 diabetes leads to significant changes in sleep breathing parameters. To minimize the confounding effect of weight loss, those subjects with a reduction of BMI ≥2.0 kg/m^2^ were excluded. 

## 2. Experimental Section

### 2.1. Statement on Ethics

The study was conducted in accordance with the ethical guidelines of the Helsinki Declaration. The human ethics committee of the Hospital Universitari Arnau de Vilanova approved the study. Informed written consent was obtained from all participants. 

### 2.2. Study Design and Description of the Study Population

This prospective interventional study assessed the consequences of improving glycemic control on sleep breathing parameters in subjects with type 2 diabetes without any known pulmonary disease. The study examined a total of 216 consecutive Caucasian subjects with type 2 diabetes at their initial visit to the outpatient Diabetes Clinic from April 2018 to September 2019 (Figure S1).

The inclusion criteria included: aged between 40 and 70 years, a BMI lower than 40 kg/m^2^, HbA1c ≥7.5% (58 mmol/mol), no medical history of lung disease and type 2 diabetes with at least 5 years of follow-up. Among the 145 patients who met the inclusion criteria, we excluded 55 for the following reasons: an inability to understand instructions on how to conduct the study at home (*n* = 15), unwillingness to participate in the study (*n* = 14), patients under treatment with continuous positive airway pressure (CPAP) (*n* = 12), hyperglycemia secondary to corticosteroids (*n* = 9), active malignancy or malignancy diagnosed within the previous five years (*n* = 3) and heart failure (*n* = 2). No pregnant women were included in the study. Finally, a baseline sleep study was performed at home in 90 subjects, and only those with baseline AHI ≥ 5 events/hour and studies with more than 5 hours of data register (*n* = 68) were invited to repeat the study after a 3-month period, during which antidiabetic treatment was strengthened. Nine patients failed to perform the second evaluation. In addition, to minimize the influence of weight loss on the results, 14 patients who reduced their BMI by more than 2.0 kg/m^2^ were excluded, as well as 10 patients with less than 5 hours of data registered. In all, 35 patients were included in the study. Those with a reduction of their HbA1c ≥0.5% (arbitrary set point) were considered good responders (*n* = 24) and the other 11 non-responders, patients. Total body fat and abdominal fat were estimated using the Body Adiposity Estimator (CUN-BAE) and the equation proposed by Bonora et al., respectively [11,12]. A control group of 9 healthy subjects, without type 2 diabetes or lung disease, was recruited from May 2019 to September 2019 among the relatives of patients with diabetes, as well as the employees of our institution. 

### 2.3. Sleep Breathing Measurements

All participants underwent an overnight home sleep study using non-attended cardiorespiratory polygraphy (Embletta; ResMed, Madrid, Spain) according to standard techniques. The recorded parameters included oronasal flow, thoracoabdominal movements, electrocardiogram and pulse oximetry. A trained scorer blinded to the study reviewed all sleep studies manually. An apnea was defined as a decrease of 90% in pre-event baseline airflow with a duration of at least 10 seconds. Hypopnea was defined as a ≥30% reduction in nasal cannula tracing with respect to baseline with a duration of at least 10 seconds and associated with a drop in arterial oxygen saturation (SaO_2_) ≥ 3%. The AHI was characterized as the sum of apneas plus hypopneas recorded during the study per hour of monitoring time. In accordance with the American Academy of Sleep Medicine scoring criteria, obstructive sleep apnea (OSA) was defined as an AHI ≥ 5 events/hour, and patients were divided into non-OSA (AHI < 5 events/hour), mild OSA (AHI between 5 and 15 events/hour), moderate OSA (AHI between 15 and 30 events/hour), and severe OSA (AHI >30 events/hour) [13]. Five oxygen saturation measures were assessed: the cumulative percentage of time spent with oxygen saturations below 90% (CT90), the oxygen desaturation index at 3% (ODI 3%), and the baseline, median and the minimum SpO_2_ levels. The degree of sleepiness was evaluated using the Epworth Sleepiness Scale (ESS), a widely used questionnaire based on the tendency to fall asleep during various daytime situations [14]. A score of 10 or more is considered sleepy.

### 2.4. Type 2 Diabetes Treatment at Baseline and during Glycemic Intensification

Type 2 diabetes was defined according to the criteria recommended by the Expert Committee on the Diagnosis and Classification of Diabetes [15]. At baseline evaluation, patients were under treatment with metformin alone (11.4%), metformin plus other oral agents (22.9%), basal insulin alone or with basal-bolus regimen (25.7%), glucagon-like peptide-1 (GLP-1) receptor agonists plus oral agents (5.7%) and basal insulin associated with GLP-1 (8.6%). All participants followed a balanced and normocaloric diet with moderate amounts of carbohydrate (45% of total energy intake, approximately). No patient was treated with diet alone.

All subjects underwent treatment intensification to improve glycemic control according to our routine medical practice. At the end of the study, the proportion of patients receiving insulin therapy had increased to 37.8% and 51.4% were treated with GLP-1 receptor agonists (12 of the 18 patients in combination with insulin) and 11.4% were on oral therapy only. None of the subjects remained on treatments comprising only diet or metformin.

Fasting plasma glucose (hexoquinase method (Olympus Diagnostica GmbH, Hamburg, Germany)) and HbA1c (chromatography) were obtained from all patients with type 2 diabetes.

### 2.5. Statistical Analysis 

The SPSS software (IBM SPSS Statistics for Windows, Version 20.0. Armonk, NY, USA) was used to perform the statistical analyses. A normal distribution of the variables was established using the Kolmogorov–Smirnov test. Data were expressed as median (range), mean ± SD or as a percentage. The χ^2^ test was used to compare changes in categorical variables between baseline and the end of the follow-up, while a paired Student’s t-test was used to compare non categorical data. The Kruskal–Wallis test and the one-way analysis of variance were used to compare both nonparametric and parametric variables between more than two groups. The relationship between continuous variables was examined by the Pearson linear correlation test. A stepwise multivariate regression analysis was performed to explore the variables independently related to the absolute change of AHI and the minimum SaO_2_. Variables significantly associated with changes in sleep breathing parameters in the bivariate analysis (i.e., absolute change in HbA1c), together with clinically relevant variables with a potential impact on sleep breathing dysfunction (i.e., age, gender, baseline and absolute change in BMI, smoking status (never smokers vs. former and past smokers)) and type 2 diabetes chronic complications (baseline HbA1c and known type 2 diabetes duration) were included in the analysis. All “*p*” values were based on a two-sided test of statistical significance, and significance was accepted at a level of *p* < 0.05.

## 3. Results

After a follow-up period of 113.2 ± 10.2 days, 24 patients (68.5%) were considered good responders. In this group, HbA1c significantly decreased from 8.8 ± 0.9 to 7.3 ± 0.6 % (73.4 ± 9.9 to 56.9 ± 6.9 mmol/mol, *p* < 0.001) (Table 1). By contrast, 11 patients (31.4%) were classified as non-responders, with a mean change in HbA1c of 0.3% (95% CI: −0.0 to 0.7). No differences at baseline were observed between the groups (good responders and non-responders) regarding clinical, metabolic or nocturnal breathing parameters (Table S1).

Subjects who exhibited a significant improvement in their metabolic control also revealed a positive and significant impact in their AHI (26.1 (8.6 to 95.0) events/hour at baseline vs. 20.0 (4.0 to 62.4) events/hour at the end of the study, *p* = 0.002), CT90 (13.3 (0.4 to 69.0) % at baseline vs. 8.1 (0.4 to 71.2) % at the end of the study, *p* = 0.002) and ODI 3% (37.6 ± 21.2 vs. 28.6 ± 19.5, *p* < 0.001) (Table 1). These changes were achieved without significant changes in BMI, waist and neck circumference, estimated total body fat and abdominal adiposity during the follow-up period. Similar results were observed when nocturnal sleep breathing parameters were assessed in the entire population but disappeared when the group of non-responders was evaluated. In the control group, AHI and CT90 values did not change between baseline and after a follow-up period of 151.9 ± 35.1 days (AHI: 15.1 (6.3 to 25.9) vs. 15.3 (3.2 to 51.5) events/hour, *p* = 0.594; CT90: 0.2 (0.0 to 2.1) vs. 0.4 (0.0 to 15.0) %, *p* = 0.093), similar to the group of non-responders.

**Figure 1:**
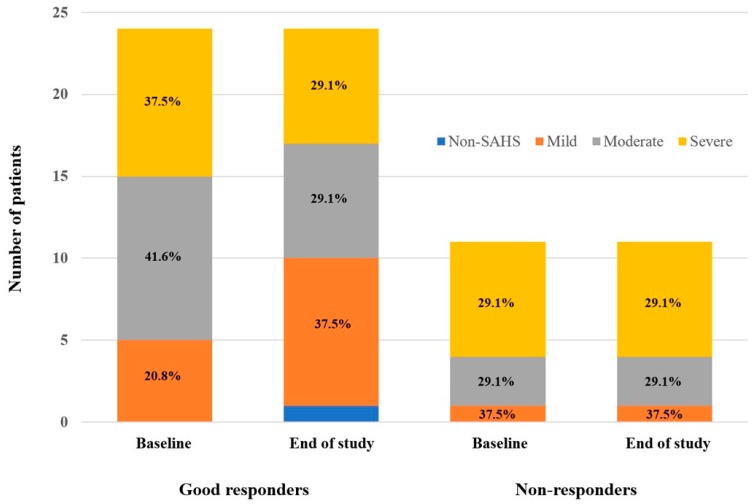
Distribution of the sleep apnea hypopnea syndrome gradation in patients with type 2 diabetes included in the study according to the group (good responders and non-responders) at baseline and after the intensification period. Non-OSA: apnea–hypopnea index (AHI) < 5 events/hour; mild OSA: AHI between 5 and 15 events/hour; moderate OSA: AHI between 15 and 30 events/hour; severe OSA (AHI >30 events/hour).

From the baseline to the end of the study, the percentage of patients with a mild OSA increased from 20.8% to 37.5% in the group of good responders, whereas the percentage of moderate and severe OSA decreased from 41.6% and 37.5% to 29.1% and 29.1%, respectively (*p* = 0.040) (Figure 1). However, no changes in the number of patients with type 2 diabetes in each classification of OSA occurred among the non-responder group.

At the end of follow-up, 13 patients were under treatment with insulin treatment, 6 were on GLP-1 receptor agonist, and 12 received treatment with insulin plus a GLP-1 receptor agonist. The nocturnal sleep measurements at baseline and after the metabolic improvement period were similar between these three groups (Table S2). However, those patients treated with insulin alone obtained better results in terms of AHI and CT90 at the end of the study (Table S3).

**Figure 2:**
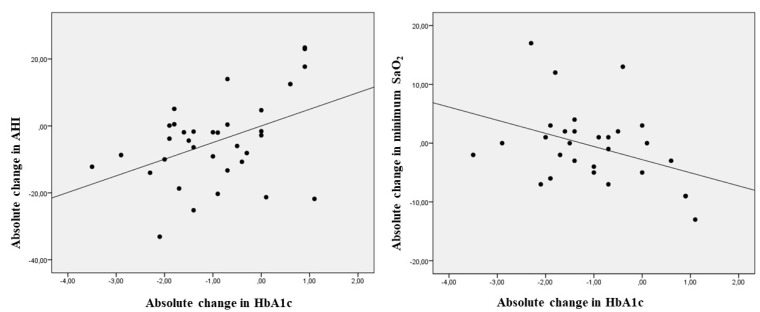
Scatter plot showing the linear correlation between absolute changes in HbA1c and absolute changes in the apnea hypoapnea index and the minimum arterial oxygen saturation during register time. AHI: apnea hypoapnea index; SaO_2_: arterial oxygen saturation; HbA1c; glycated hemoglobin.

In the entire population, univariate analysis showed that the absolute decrease in HbA1c from baseline to the end of the study correlated to significant decrements in the AHI (r = 0.358, *p* = 0.035) and increments in the minimum SaO_2_ achieved during the nocturnal time (r = −0.386, *p* = 0.039) (Figure 2). The other correlations observed in the univariate analysis are displayed in Table S4. 

Finally, the stepwise multivariate regression analysis performed in the entire population with type 2 diabetes showed that baseline AHI together with the absolute change in HbA1c (but not baseline HbA1c, age, gender, known years with type 2 diabetes, smoking status, baseline BMI and absolute change in the BMI) independently predicted changes in the AHI (R^2^ = 0.496) (Table 2). In addition, baseline values of HbA1c and AHI independently predicted changes in the minimum SaO_2_ (R^2^ = 0.288).

## 4. Discussion

To the best of our knowledge, this is the first study to offer evidence that nocturnal sleep breathing parameters exhibit significant amelioration after the mid-term improvement of glycemic control in patients with type 2 diabetes. This favorable change in the sleep breathing data was only observed among good responders, that is to say, subjects who reduced their HbA1c by more than 0.5%. Therefore, our study reinforces the notion that type 2 diabetes exerts a negative impact on sleep breathing that is tightly linked to metabolic control. Remarkably, the most sensitive parameter associated with the enhancement of glycemic control is related to reductions in the AHI. It should be noted that in our study, the group of good responders experienced a 23.3% reduction in their AHI after almost 4 months of metabolic improvement. As OSA significantly elevates the risk of microangiopathy, kidney disease, coronary heart disease and all-cause mortality in patients with type 2 diabetes in comparison with non-diabetic individuals, it seems reasonable to postulate positive clinical consequences for this intervention [16,17]. In addition, our results might provide new insights into how metabolic control is associated with the prevention and amelioration of diabetic complications.

Although 97% of clinicians agreed that OSA is prevalent in subjects with type 2 diabetes, the negative impact that type 2 diabetes exerts on sleep breathing is still under-recognized [18]. Truthfully, OSA is highly prevalent among patients with type 2 diabetes in both clinic-based and community-based cohorts, with an overall prevalence exceeding 50% [19,20]. In addition, patients with type 2 diabetes also exhibit an increased incidence of OSA compared with the general population. In this way, when data from 151,194 participants without OSA from three ongoing prospective cohort studies in the United States was analyzed, the hazard ratio for developing OSA was discreetly higher in individuals with diabetes (1.08 (95% CI: 1.00 to 1.16), but increased substantially among those subjects under insulin treatment compared with those without diabetes (1.43 (95% CI: 1.11 to 1.83)) [21]. Different pathogenic mechanisms have been suggested as contributing to the higher incidence of sleep breathing disorders among patients with type 2 diabetes: insulin resistance, leptin resistance, systemic inflammation and autonomic or peripheral neuropathy [1,22]. Intriguingly, this marked vulnerability to OSA in type 2 diabetes is remarkably higher in women than in men with diabetes, with the implicated sex-specific mechanisms still to be elucidated [21,23].

Although intermittent hypoxia and sleep fragmentation, the two hallmarks of OSA, have been associated with insulin resistance and higher rates of type 2 diabetes, the effect of CPAP treatment on insulin resistance and glycemic control is conflicting [1,17,23]. Two recent meta-analyses involving 1077 participants with type 2 diabetes showed no effectiveness regarding changes in HbA1c, fasting glucose or fasting insulin levels after 12 or 24 weeks of CPAP treatment [24,25]. A third meta-analysis evaluated the effect of CPAP on 443 participants without diabetes, showing significant improvements in insulin resistance measured with the HOMA-index without any impact on fasting glucose [26]. However, although a potential bi-directionality between type 2 diabetes and OSA is constantly proposed, the effective treatment of diabetes appears more likely to provoke serious consequences on intermittent hypoxia than vice versa. In this way, our study also confirms and expands on previous data from our group in which only a five-day period of blood glucose optimization produced a significant reduction in the increased number of nocturnal oxygen desaturations [9].

The way in which metabolic control was improved in our study included different antidiabetic drugs, and this condition must be considered when our results are evaluated. In experimental models, the valuable effects of metformin and insulin on sleep breathing have been observed. Thus, in insulin resistant Sprage-Dawley rats, oral treatment with metformin not only prevented, but also reversed apnea episodes [27]. Similarly, in streptozotocin-induced diabetic rats insulin treatment prevented the reduction in ventilatory responses to hypercapnic and hypoxic challenges described at the end of the first months of life [28]. The role of insulin-sensitizer therapies on nocturnal sleep breathing in our study appears small because neither metformin nor thiazolidinediones were added during follow-up. The underlying deficit of GLP-1 in type 2 diabetes could also be involved in the impairment of sleep breathing, and the potential benefit of incretin-based therapies needs to be evaluated (ClinicalTrials.gov: NCT02889510). After 32 weeks of treatment, daily liraglutide 3.0 mg administered to obese patients with moderate to severe OSA produced a greater mean percentage reduction of the AHI compared with placebo (−12.2 *vs*. −6.6 events/hour, *p* = 0.015) [29]. However, this amelioration in AHI was closely related with weight loss. Similarly, a reduction in AHI and an increase in minimum SaO_2_ were detected in newly diagnosed patients with type 2 diabetes and OSA who were treated for 24 weeks with dapagliflozine, a sodium glucose co-transporter 2 inhibitor, but not in the placebo group [30]. Our study has tried to minimize the beneficial effect of weight loss on nighttime breathing by excluding those patients who experienced a significant reduction in their BMI between the baseline study and the end of the follow-up. In addition, estimated total body and abdominal adiposity was not modified during the follow-up period, in either the good responder or the non-responder groups. In the multivariate regression analysis the absolute change in HbA1c, but not modifications in the BMI, independently predicted decrements in AHI. With this, we provide strong evidence that the improvement of glycemic control by itself exerts a positive impact on respiratory function, rather than the therapeutic option used to achieve it.

The most cost-effective manner of screening sleep breathing abnormalities in type 2 diabetes is a key issue. Appropriate studies to determine whether simple tools such as questionnaires should be included in routine visits to evaluate daytime sleepiness and sleep quality are needed. The administration of the STOP-BANG (Snoring, Tired, Observed, Pressure, BMI, Age, Neck and Gender) questionnaire was able to identify 90.6% of patients with type 2 diabetes and OSA [31]. Recently, a French panel of endocrinologists and pneumologists justified screening for OSA in patients with type 2 diabetes and clinical symptoms, and even with no symptoms in those subjects with microvascular complications or uncontrolled blood pressure [32]. Our results, in which the amelioration of sleep breathing parameters was not related to the Epworth score support the hypothesis that screening should be generalized to all patients. Together with questionnaires, neck circumference and neck grasp are also useful for identifying patients with type 2 diabetes who are at risk of OSA in the primary care setting [33]. However, there is still a long way to go before we have biological markers that can help us in this matter [34].

There are certain potential constraints that should be judged in evaluating the results of our study. First, we evaluated a relatively small number of patients with type 2 diabetes with different degrees of OSA, which means that no definite consequences for daily clinical practice can be extrapolated to the general population with type 2 diabetes. Nevertheless, the participants were selected with extreme care to avoid confounding factors associated with sleep breathing such as changes in body weight or body composition. In addition, a control group of non-responders was also considered, as well as a group of subjects without diabetes. Second, we only present evidence about the positive effects of metabolic improvement after a relative short period (4 months). Therefore, larger studies with a longer follow-up are needed to confirm and extend our results, as well as to go deeper into the potential impact of different antidiabetic drugs on nocturnal sleep breathing. Finally, nocturnal sleep breathing was assessed by home sleep testing. Home respiratory polygraphic recordings underscore the AHI, mostly because the total recording time and not the total sleep time is used as the denominator when calculating the AHI. However, home sleep studies have been shown to be a valid alternative to laboratory-based polysomnography [35]. In any case, our study did not aim to establish an accurate diagnosis of OSA in patients with type 2 diabetes, but to evaluate changes from baseline following improvements in metabolic control.

## 5. Conclusions

In conclusion, an improvement in glycemic control was associated with positive changes in sleep breathing parameters in patients with type 2 diabetes. The main impact was related to significant reductions in the AHI that were independent of weight modifications. Our results confirm the idea that the lung should be considered a target of diabetic complications.

## Figures and Tables

**Table 1:** Evolution of the main nocturnal sleep breathing parameters according to the response to the intensification of the antidiabetic treatment.

	Baseline	End of Study	Mean Difference (95% CI)	*p*
**Entire population**				
*n*	35	35	-	-
AHI (events per hour)	28.5 (6.5 to 95.0)	24.0 (4.0 to 62.4)	-	0.022
CT90 (%)	12.0 (0.0 to 87.8)	8.2 (0.0 to 71.2)	-	<0.001
Epworth	5.7 ± 3.5	4.9 ± 2.8	−0.8 (−1.4 to −0.1)	0.018
ODI 3% (events per hour)	40.3 ± 21.9	33.7 ± 22.1	−6.5 (−11.2 to −1.8)	0.007
Baseline SaO_2_ (%)	97.8 ± 1.2	98.1 ± 1.4	0.3 (−0.4 to 1.0)	0.477
Average SaO_2_ (%)	91.4 ± 2.1	91.6 ± 2.4	0.2 (−0.4 to 0.9)	0.674
Minimum SaO_2_ (%)	75.5 ± 9.5	75.0 ± 12.0	−0.5 (−3.0 to 1.9)	0,374
HbA1c (%)	8.8 ± 0.9	7.8 ± 1.0	−0.9 (−1.3 to −0.5)	<0.001
HbA1c (mmol/mol)	72.7 ± 10.1	62.5 ± 11.2	−10.1 (−14.2 to −6.0)	<0.001
BMI (kg/m^2^)	35.1 ± 4.5	35.0 ± 4.5	−0.05 (−0.2 to 0.1)	0.665
Waist circumference (cm)	116.3 ± 12.4	116.2 ± 12.3	−0.0 (−1.3 to 1.1)	0.892
Neck circumference (cm)	41.9 ± 3.8	41.7 ± 3.9	−0.2 (−0.5 to 0.1)	0.188
CUN−BAE (%)	41.4 ± 6.7	41.4 ± 6.7	−0.0 (−0.3 to 0.2)	0.705
Bonora equation (cm^2^)	272.4 ± 79.4	273.9 ± 80.4	1.5 (−5.2 to 8.2)	0.652
**Good responders**				
*n*	24	24	-	-
AHI (events/hour)	26.1 (8.6 to 95.0)	20.0 (4.0 to 62.4)	-	0.002
CT90 (%)	13.3 (0.4 to 69.0)	8.1 (0.4 to 71.2)	-	0.002
ODI 3% (events per hour)	37.6 ± 21.2	28.6 ± 19.5	−10.1 (−14.9 to −5.3)	<0.001
Epworth	5.4 ± 3.1	4.7 ± 2.5	−0.7 (−1.6 to 0.1)	0.083
Baseline SaO_2_ (%)	97.8 ± 1.1	98.0 ± 1.6	0.2 (−0.7 to 1.2)	0.565
Average SaO_2_ (%)	91.2 ± 1.9	91.4 ± 2.4	0.2 (−0.7 to 1.1)	0.592
Minimum SaO_2_ (%)	76.3 ± 10.2	76.7 ± 11.3	0.3 (−2.2 to 3.0)	0.765
HbA1c (%)	8.8 ± 0.9	7.3 ± 0.6	−1.5 (−1.8 to −1.2)	<0.001
HbA1c (mmol/mol)	73.4 ± 9.9	56.9 ± 6.9	−16.5 (−19.8 to −13.1)	<0.001
BMI (kg/m^2^)	34.5 ± 4.6	34.4 ± 4.6	−0.1 (−0.4 to 0.2)	0.504
Waist circumference (cm)	115.7 ± 12.8	115.7 ± 12.8	0.0 (−1.1 to 1.1)	0.994
Neck circumference (cm)	41.4 ± 4.0	41.2 ± 4.1	−0.1 (−0.5 to 0.2)	0.366
CUN-BAE (%)	40.9 ± 6.6	40.8 ± 6.7	−0.1 (−0.4 to 0.2)	0.568
Bonora equation (cm^2^)	270.7 ± 80.5	273.9 ± 80.4	1.5 (−5.2 to 8.2)	0.652
**Non-responders**				
*n*	11	11	-	-
AHI (events/hour)	31.4 (6.5 to 63.2)	41.4 (5.2 to 58.5)	-	0.722
CT90 (%)	11.8 (0.0 to 87.8)	10.9 (0.0 to 51.5)	-	0.138
ODI 3% (events per hour)	43.6 ± 24.0	44.6 ± 24.2	−0.0 (−9.0 to 11.0)	0.839
Epworth	6.1 ± 4.1	5.2 ± 3.4	−0.9 (−2.1 to 0.3)	0.127
Baseline SaO_2_ (%)	98.0 ± 1.5	98.4 ± 0.7	0.4 (−0.9 to 1.8)	0.482
Average SaO_2_ (%)	92.0 ± 2.7	92.2 ± 2.3	0.2 (−0.7 to 1.2)	0.563
Minimum SaO_2_ (%)	73.3 ± 7.8	70.5 ± 13.5	−2.8 (−9.7 to 4.0)	0.357
HbA1c (%)	8.6 ± 0.9	8.9 ± 0.8	0.3 (−0.0 to 0.7)	0.061
HbA1c (mmol/mol)	71.0 ± 10.7	74.7 ± 9.1	3.6 (−0.4 to 7.7)	0.078
BMI (kg/m^2^)	36.3 ± 4.3	36.4± 4.3	0.0 (−0.3 to 0.4)	0.725
Waist circumference (cm)	117.4 ± 12.2	117.1 ± 11.7	−0.2 (−3.7 to 3.1)	0.864
Neck circumference (cm)	43.1 ± 3.3	42.7 ± 3.4	−0.3 (−1.2 to 0.4)	0.363
CUN-BAE (%)	42.5 ± 7.0	42.5 ± 6.8	0.0 (−0.3 to 0.4)	0.726
Bonora equation (cm^2^)	275.9 ± 80.7	277.2 ± 79.9	1.3 (−17.0 to 19.7)	0.875

Data are means ± SD or *n* (percentage). AHI: apnea hypopnea index; CT90: registered time with oxygen saturation below 90%; ODI 3%: oxygen desaturation index at 3%; SaO2: arterial oxygen saturation; HbA1c; glycated hemoglobin; BMI: body mass index; CUN-BAE: Clínica Universidad de Navarra-Body Adiposity Estimator.

**Table 2:** Variables independently related to changes in the apnea–hypopnea index and minimum SaO_2_ in the multiple regression analysis (stepwise method).

	β	Beta 95% CI	*p*
**∆ AHI**			
Baseline AHI (events/hour)	−0.614	−0.443 (−0.630 to −0.256)	<0.001
∆ HbA1c (%)	0.453	6.565 (2.814 to 10.315)	0.001
Baseline HbA1c (%)	−0.189	-	0.193
Baseline BMI	0.058	-	0.448
Age (yrs)	0.057	-	0.677
Smoking status *	0.033	-	0.811
∆ BMI (kg/m^2^)	−0.023	-	0.865
Known type 2 diabetes duration (yrs)	−0.015	-	0.915
Gender	0.009	-	0.949
Constant	-	15.197 (6.403 to 23.991)	0.001
R^2^ = 0.496			
**∆ mínimum SaO_2_**			
Baseline HbA1c (%)	0.360	2.378 (0.116 to 4.639)	0.040
Baseline AHI (events/hour)	−0.355	−0.118 (−0.232 to −0.004)	0.043
Gender	−0.224	-	0.241
Age (yrs)	−0.166	-	0.367
∆ AHI	−0.166	-	0.406
Known type 2 diabetes duration (yrs)	−0.150	-	0.427
Smoking status *	−0.124	-	0.504
∆ HbA1c (%)	−0.134	-	0.535
∆ BMI (kg/m^2^)	−0.059	-	0.735
Baseline BMI	0.031	-	0.860
Constant	-	−17.386 (−38.175 to 3.403)	0.097
R^2^ = 0.288			

β: standardized coefficient; Beta: non-standardized coefficient; ∆: absolute change; AHI: apnea–hypopnea index; HbA1c: glycated hemoglobin; SaO_2_: arterial oxygen saturation; BMI: body mass index; CI: confidence interval. * never smokers vs. former and past smokers.

## Supplementary Materials

The following are available online at https://www.mdpi.com/2077-0383/9/4/1022/s1, Figure S1: Flow chart of the study population, Table S1: Baseline main clinical, metabolic and nocturnal sleep breathing characteristics of participants in the study according to their response to the intensification of antidiabetic treatment, Table S2: Characteristics of the main nocturnal sleep breathing parameters according to the antidiabetic treatment (insulin or glucagon like peptide 1 receptor analogue) prescribed at the end of the follow-up period, Table S3: Evolution of the main nocturnal sleep breathing parameters according to the antidiabetic treatment (insulin or glucagon like peptide 1 receptor analogue) prescribed at the end of the follow-up period, Table S4: Correlations of clinical variables with absolute changes in the nocturnal sleep breathing parameters in participants with type 2 diabetes.
