# Supplementary material for: Effect of Glucose Improvement on Nocturnal Sleep Breathing Parameters in Patients with Type 2 Diabetes: The Candy Dreams Study
Source: J Clin Med. 2020 Apr 4;9(4):1022. doi: 10.3390/jcm9041022 (PMC7230160; doi:10.3390/jcm9041022)
Supplement: Supplementary file 1 [file jcm-09-01022-s001.pdf]

Supplemental Figure 1. Flow chart of the study population.

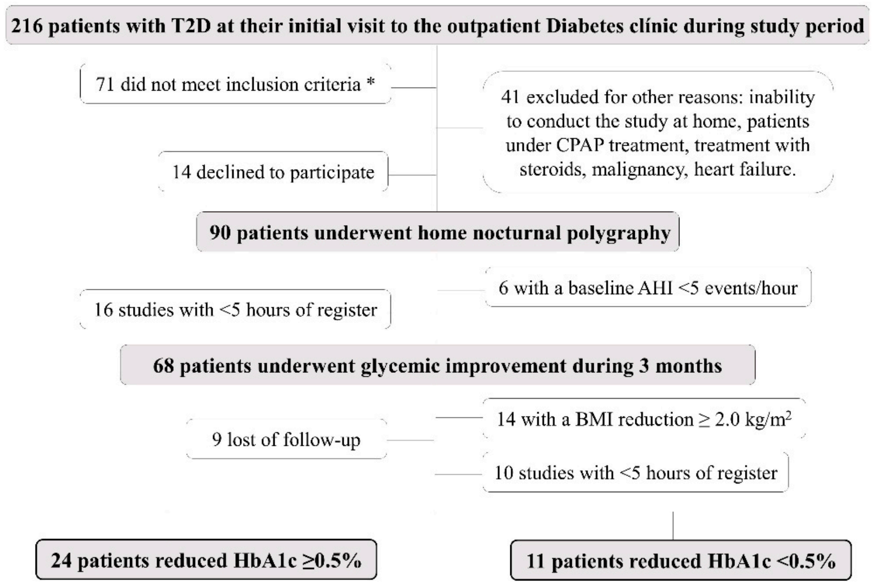

The inclusion criteria included: age between 40 and 70 years old, BMI lower than 40 kg/m^2, HbA1c ≥7.5% (58 mmol/mol), absence of medical history of lung disease, and T2D with at least 5 years of follow-up. T2D: type 2 diabetes; CPAP: continuous positive airway pressure; AHI: apnea-hypopnea index; BMI: body mass index; HbA1c: glycated hemoglobin..

Figure S1: Flow chart of the study population

Supplemental Table S1. Baseline main clinical, metabolic and nocturnal sleep breathing characteristics of participants in the study according to their response to the intensification of antidiabetic treatment.

	**All patients**	**Good responders**	**Non-responders**	**p**
n	35	24	11	-
**Clinical data**				
Men, n (%)	22 (62.8)	15 (62.5)	7 (63.6)	0.626
Age (yrs)	55.6 ± 7.4	56.5 ± 7.1	53.6 ± 8.2	0.294
BMI (Kg/m^2)	35.1 ± 4.5	34.5 ± 4.6	36.3 ± 4.3	0.282
Decrease in BMI (kg/m^2)	-0.15 ± 0.7	-0.1 ± 0.7	0.0 ± 0.5	0.518
Decrease in Kg (kg)	-0.01 ± 2.0	-0.3 ± 2.1	-0.2 ± 1.6	0.394
Waist circumference (cm)	116.3 ± 12.4	115.7 ± 12.8	117.4 ± 12.2	0.727
Neck circumference (cm)	41.9 ± 3.8	41.4 ± 4.0	43.1 ± 3.3	0.232
CUN-BAE (%)	41.4 ± 6.7	40.9 ± 6.6	42.5 ± 7.0	0.538
Bonora equation (cm^2)	272.4 ± 79.4	270.7 ± 80.5	275.9 ± 80.7	0.863
Never smokers (%)	18 (51.4)	10 (41.6)	8 (72.7)	0.146
Known diabetes duration (yrs)	11.7 ± 7.8	12.1 ± 8.0	10.7 ± 7.5	0.621
Diabetic Retinopathy, n (%)	12 (34.2)	9 (25.7)	3 (30.0)	0.709
Ischemic heart disease, n (%)	5 (14.2)	3 (12.0)	2 (20.0)	0.640
**Metabolic data**				
Baseline HbA1c (%)	8.8 ± 0.9	8.8 ± 0.9	8.6 ± 0.9	0.495
Baseline HbA1c (mmol/mol)	72.7 ± 10.1	73.4 ± 9.9	71.0 ± 10.7	0.529
Decrease in HbA1c (%)	-0.9 ± 1.1	-1.5 ± 0.7	0.3 ± 0.5	<0.001
Decrease in HbA1c (mmol/mol)	-10.1 ± 12.0	-16.5 ± 8.0	3.6 ± 6.1	<0.001

FPG (mmol/l)	195.1 ± 68.6	188.5 ± 66.0	209.4 ± 75.1	0.411
Triglycerides (mg/dl)	183.0 (72.0-701.0)	142.0 (72.0-366.0)	183.0 (82.0-701.0)	0.540
LDL-cholesterol (mg/dl)	114.9 ± 35.2	112.3 ± 38.5	120.4 ± 28.2	0.541
ACR (mg/g)	79.9 ± 16.3	78.9 ± 16.5	81.3 ± 16.3	0.582
**<u>Nocturnal sleep breathing data</u>**				
AHI (events per hour)	28.5 (6.5 to 95.0)	26.1 (8.6 to 95.0)	31.4 (6.5 to 63.2)	0.334
CT90 (%)	12.0 (0.0 to 87.9)	13.3 (0.4 to 69.0)	11.8 (0.0 to 87.8)	0.847
Baseline SaO_2 (%)	97.8 ± 1.1	97.7 ± 1.0	98.0 ± 1.5	0.611
ODI 3% (events per hour)	40.3 ± 24.0	37.6 ± 21.2	40.3 ± 21.9	0.468
Average nocturnal SaO_2 (%)	91.4 ± 2.1	91.2 ± 1.8	92.0 ± 2.7	0.409
Minimum nocturnal SaO_2 (%)	75.3 ± 9.4	76.0 ± 10.0	73.3 ± 7.8	0.504
Epworth questionnaire	5.8 ± 3.5	5.7 ± 3.3	6.1 ± 4.1	0.733

Data are mean ± SD, median (range) or n (percentage). BMI: body mass index; FPG: fasting plasma glucose; LDL: low density lipoprotein; ACR: albuminuria-to-creatinine ratio; SaO_2: arterial oxygen saturation ()

Supplemental Table S2. Characteristics of the main nocturnal sleep breathing parameters according to the antidiabetic treatment (insulin or glucagon like peptide 1 receptor analogue) prescribed at the end of the follow-up period.

	**Insulin**	**GLP-1ra**	**Insulin plus GLP-1ra**	**p ANOVA**
n	13	6	~~11~~ 12	-
**<u>Baseline</u>**				
HbA1c (%)	9.2 ± 1.0	8.7 ± 0.8	8.5 ± 0.7	0.190
HbA1c (mmol/mol)	77.3 ± 11.1	72.1 ± 9.5	69.1 ± 8.1	0.187
Epworth	5.8 ± 3.1	7.1 ± 4.6	5.5 ± 3.8	0.688
AHI (events/hour)	28.5 (8.6 to 63.2)	25.8 (6.5 to 44.6)	30.9 (10.4 to 79.0)	0.676
CT90 (%)	9.5 (0.0 to 61.0)	10.4 (5.5 to 31.0)	14.3 (0.4 to 87.8)	0.727
ODI 3% (events per hour)	43.5 ± 25.8	29.2 ± 18.7	42.9 ± 21.6	0.421
Baseline SaO_2 (%)	98.2 ± 1.0	98.0 ± 0.0	97.6 ± 1.4	0.578
Average SaO_2 (%)	92.1 ± 2.0	91.0 ± 1.0	90.6 ± 2.3	0.229
Minimum SaO_2 (%)	80.1 ± 4.9	69.3 ± 8.6	69.8 ± 11.4	0.019
**<u>End of study</u>**				
HbA1c (%)	8.2 ±1.0	7.9 ± 1.4	7.6 ± 0.7	0.391
HbA1c (mmol/mol)	66.7 ± 10.9	63.8 ± 15.9	60.0 ± 15.9	0.356
Epworth	5.6 ± 2.9	4.6 ± 2.8	4.6 ± 2.9	0.687
AHI (events/hour)	21.3 (5.2 to 62.4)	20.3 (11.2 to 49.3)	34.2 (4.0 to 62.1)	0.586
CT90 (%)	8.2 (0.0 to 36.0)	5.9 (0.4 to 10.0)	10.0 (0.5 to 71.2)	0.234
ODI 3% (events per hour)	38.9 ± 26.5	23.8 ± 16.1	39.7 ± 20.1	0.331
Baseline SaO_2 (%)	97.4 ± 1.4	98.0 ± 1.2	98.4 ± 1.2	0.244
Average SaO_2 (%)	92.0 ± 2.9	92.4 ± 0.8	91.1 ± 2.5	0.590
Minimum SaO_2 (%)	80.5 ± 7.2	72.8 ± 13.4	67.6 ± 13.6	0.045

HbA1c: glycated hemoglobin; AHI: apnea-hypopnea index; CT90: time of register with oxygen arterial saturation below 90%; SaO_2: arterial oxygen saturation.

Supplemental Table 3. Evolution of the main nocturnal sleep breathing parameters according to the antidiabetic treatment (insulin or glucagon like peptide 1 receptor analogue) prescribed at the end of the follow-up period.

	**Baseline**	**End of study**	**Mean difference (95% CI)**	**p**
**Insulin**				
n	13	13	-	-
Good responders, n (%)	-	9 (69.2)	-	-
HbA1c (%)	9.2 ± 1.0	8.2 ± 1.0	-0.9 (-1.5 to -0.3)	0.007
HbA1c (mmol/mol)	77.3 ± 11.1	66.7 ± 10.9	-10.5 (-17.5 to -3.4)	0.007
BMI (kg/m^2)	31.5 ± 3.2	31.6 ± 3.0	0.1 (-0.3 to 0.5)	0.652
Epworth	5.8 ± 3.1	5.6 ± 2.9	-0.1 (-0.9 to 0.6)	0.617
AHI (events/hour)	28.5 (8.6 to 63.2)	21.3 (5.2 to 62.4)	-	0.003
CT90 (%)	9.5 (0.0 to 61.0)	8.2 (0.0 to 36.0)	-	0.047
ODI 3% (events per hour)	46.1 ± 25.1	38.9 ± 26.5	-7.2 (-12.9 to -1.4)	0.019
Baseline SaO_2 (%)	98.5 ± 1.0	97.3 ± 1.7	-1.1 (-2.7 to 0.3)	0.110
Average SaO_2 (%)	92.1 ± 2.1	92.0 ± 2.9	-0.09 (-1.3 to 1.1)	0.878
Minimum SaO_2 (%)	81.0 ± 3.9	80.5 ± 7.2	-0.5 (-4.7 to 3.6)	0.780
**arGLP1**				
n	6	6	-	-
Good responders, n (%)	-	3 (50.0)	-	-
HbA1c (%)	8.7 ± 0.8	7.9 ± 1.4	-0.7 (-2.0 to -0.5)	0.192
HbA1c (mmol/mol)	70.2 ± 7.9	60.2 ± 8.1	-10.0 (-17.9 to -2.0)	0.192
BMI (kg/m^2)	39.3 ± 3.7	39.1 ± 4.2	-0.1 (-1.0 to 0.6)	0.582
Epworth	6.4 ± 4.7	4.6 ± 2.8	-1.8 (-4.6 to 1.0)	0.152
AHI (events/hour)	25.8 (6.5 to 44.6)	20.3 (11.2 to 49.3)	-	0.917
CT90 (%)	10.4 (5.5 to 31.0)	5.9 (0.4 to 10.0)	-	0.116
ODI 3% (events per hour)	29.2 ± 18.7	23.8 ± 16.1	-5.3 (-21.5 to 10.7)	0.431
Baseline SaO_2 (%)	98.0 ± 0.0	98.5 ± 2.1	0.5 (-18.5 to 19.5)	0.795
Average SaO_2 (%)	91.0 ± 1.0	92.6 ± 0.5	1.6 (-1.2 to 4.5)	0.130
Minimum SaO_2 (%)	69.3 ± 8.6	74.6 ± 17.3	0.5 (-22.1 to 32.8)	0.491
**Insulin plus arGLP1**				
n	12	12	-	-
Good responders, n (%)	-	9 (75.0)	-	-
HbA1c (%)	8.5 ± 0.7	7.6 ± 0.7	-0.9 (-1.6 to -0.2)	0.017
HbA1c (mmol/mol)	70.2 ± 7.9	60.2 ± 8.1	-10.0 (-17.9 to -2.0)	0.017
BMI (kg/m^2)	36.8 ± 3.1	36.7 ± 2.9	-0.0 (-0.3 to 0.1)	0.510
Epworth	5.4 ± 3.6	4.5 ± 2.8	-0.8 (-2.1 to 0.4)	0.193
AHI (events/hour)	30.9 (10.4 to 95.0)	33.8 (4.0 to 62.1)	-	0.814
CT90 (%)	14.3 (0.4 to 87.8)	9.7 (0.5 to 7.1)	-	0.117
ODI 3% (events per hour)	42.9 ± 21.6	39.7 ± 20.1	-3.1 (-13.4 to 7.0)	0.508
Baseline SaO_2 (%)	97.6 ± 1.4	98.4 ± 1.2	0.8 (-0.1 to 1.7)	0.082
Average SaO_2 (%)	90.6 ± 2.3	91.1 ± 2.5	0.5 (-0.5 to 1.7)	0.304
Minimum SaO_2 (%)	69.8 ± 11.4	67.6 ± 13.5	-2.1 (-0.6 to 2.1)	0.286

HbA1c: glycated hemoglobin; AHI: apnea-hypopnea index; CT90: time of register with oxygen arterial saturation below 90%; SaO_2: arterial oxygen saturation; GLP-1ra: glucagon like peptide 1 receptor analogue.

Supplemental Table S4. Correlations of clinical variables with absolute changes in the nocturnal sleep breathing parameters in participants with type 2 diabetes.

	**ΔAHI (events/hour)**		**ΔCT90 (%)**		**ΔAverage SaO2 (%)**		**ΔMinimum SaO2 (%)**	
	**r**	**p**	**r**	**p**	**r**	**p**	**r**	**p**
**Age**	-0.159	0.369	0.324	0.062	-0.353	0.006	-0.323	0.087
**Baseline HbA1c (%)**	-0.126	0.476	-0.140	0.429	0.238	0.222	0.103	0.329
**Baseline BMI (kg/m^2)**	0.055	0.757	-0.001	0.995	0.177	0.360	-0.110	0.569
**Baseline WC (cm)**	-0.140	0.431	-0.112	0.528	0.108	0.576	0.006	0.975
**Baseline NC (cm)**	-0.036	0.839	-0.009	0.960	-0.057	0.779	-0.263	0.108
**Known diabetes duration (yrs)**	-0.347	0.044	0.137	0.438	-0.454	0.013	-0.324	0.086
**Δ HbA1c (%)**	0.358	0.035	0.128	0.465	-0.017	0.929	-0.386	0.039
**Δ BMI (kg/m^2)**	-0.034	0.847	-0.247	0.160	-0.038	0.845	-0.178	0.354
**Δ WC (cm)**	-0.137	0.452	-0.329	0.058	0.133	0.491	-0.190	0.324
**Δ NC (cm)**	-0.015	0.935	0.097	0.584	0.061	0.752	0.433	0.019

Δ: absolute change; AHI: apnea hypopnea index; CT90: time of register with oxygen saturation below 90%; SaO_2: arterial oxygen saturation; HbA1c: glycated hemoglobin; BMI: body mass index; WC: waist circumference; NC: neck circumference.
